# Pediatric Patients Receiving Specialized Palliative Home Care According to German Law: A Prospective Multicenter Cohort Study

**DOI:** 10.3390/children5060066

**Published:** 2018-05-31

**Authors:** Silke Nolte-Buchholtz, Boris Zernikow, Julia Wager

**Affiliations:** 1 Department of Pediatrics, University Hospital Carl Gustav Carus, Technische Universität Dresden, Fetscherstrasse 74, 01307 Dresden, Germany; 2 Department of Children’s Pain Therapy and Pediatric Palliative Care, Faculty of Health, School of Medicine, Witten/Herdecke University, 58455 Witten, Germany; boris.zernikow@kinderklinik-datteln.de or b.zernikow@kinderklinik-datteln.de (B.Z.); j.wager@deutsches-kinderschmerzzentrum.de (J.W.); 3 Paediatric Palliative Care Centre, Children’s and Adolescents’ Hospital, 45711 Datteln, Germany

**Keywords:** palliative care, pediatrics, cancer, non-cancer, home care

## Abstract

In Germany, every child with a life-limiting condition suffering from symptoms that cannot sufficiently be controlled is eligible by law for specialized pediatric palliative home care (SPPHC). It is the aim of this study to describe the demographic and clinical characteristics of children referred to SPPHC and to compare patients with cancer and non-cancer conditions. The prospective multicenter study includes data on 75 children (median age 7.7 years, 50.7% male). The majority had non-cancer conditions (72%). The most common symptoms were cognitive impairment, somatic pain, impairment in communication or swallowing difficulties. Swallowing difficulties, seizures, and spasticity occurred significantly more often in non-cancer patients (*p* < 0.01). Cancer patients received antiemetics significantly more often (permanent and on demand) than non-cancer patients (*p* < 0.01). Significantly more non-cancer patients had some type of feeding tube (57.3%) or received oxygen (33.3%) (*p* < 0.01). Central venous catheters had been fitted in 20% of the patients, mostly in cancer patients (*p* < 0.001). Tracheostomy tubes (9.3%) or ventilation (14.7%) were only used in non-cancer patients. In conclusion, patients referred to SPPHC are a diverse cohort with complex conditions including a large range of neurologically originating symptoms. The care of pediatric palliative care patients with cancer is different to the care of non-cancer patients.

## 1. Introduction

In recent years, profound developments and improvements in the field of palliative care have taken place in Germany. Since 2007, a new law has guaranteed access to specialized (tertiary) palliative home care (SPHC) for children and adults with a life-limiting disease suffering from symptoms that cannot sufficiently be controlled in primary or secondary care. Only if these conditions are fulfilled and approved by the insurance company the patient has the right to receive SPHC. According to the law, the main tasks of SPHC are counseling, coordination and provision of palliative nursing and medical care, including a 24/7 on-call service. The law also provides quality criteria for the composition of SPHC, e.g., a certified qualification of team members and a close collaboration between specialized palliative care nurses and physicians. The 24/7 on-call duty also determines a minimum number of team members. Specialized palliative home care is delivered in close cooperation with local health care professionals, such as general practitioners, pediatricians in private practice, nursing and hospice services, and local hospitals. The costs for SPHC, whether taking place in the patient’s familiar domestic surrounding, inpatient nursing facilities, or hospices until death, are covered by health insurance when prescribed by a physician and when the patient fulfills the strict criteria defined by law [1]. 

Little is known about the demographic or clinical characteristics of children who are referred to specialized pediatric palliative home care (SPPHC) services or about the reasons for referrals to those services.

In adult SPHC patients, the mean age is more than 65 years. A majority of these patients suffer from cancer (75–94%), and the main symptoms are tiredness, fatigue, pain, and loss of appetite [2,3,4].

So far, only one study in Germany has investigated the characteristics of children receiving SPHC in comparison with characteristics of adults [5]. This study was conducted in a single center for palliative medicine, with one SPHC team for adults and one for children. Compared with the adult palliative patients, the pediatric group showed a greater diversity of diseases, with a relatively small patient group suffering from cancer. The pediatric group showed distinctive patterns of symptoms and the use of specific medications other than adults. 

The aim of this prospective multicenter cohort study was to study the characteristics of patients who are referred to nine SPPHC teams in Germany. First, demographic and medical data of the pediatric patients were described. Second, care goals were analyzed. Furthermore, the study aimed to identify differences between pediatric non-cancer and cancer patients regarding symptoms, treatment measures, and goals of consultation.

## 2. Materials and Methods

The multicenter study was carried out between April 2013 and September 2013 in nine of fourteen SPPHC teams in Germany.

### 2.1. Recruitment and Data Collection

A questionnaire was sent to the participating SPPHC teams. At each site, data on new referrals were obtained from patients’ medical charts over a time-period of three months. Before study participation, families received written study information. Patients were only included in the study if informed consent was provided by the parents. Additionally, patients able to communicate were informed about the study and asked for their assent.

Once the questionnaires were completed by the SPPHC team, they were sent back to the coordinating principal investigator at the University Hospital for children and adolescents in Dresden, Germany. The principal investigator compiled a master database and entered all data from the questionnaires into the database.

Information on the following categories was retrieved by the SPPHC teams for the time of referral:

1. Demographic data: patient age, gender, migration background, patient residence, siblings with life-limiting conditions.
2. Clinical information: current symptoms, medication, medical devices, non-pharmacological treatment.
3. Characteristics of the referral: principal underlying diagnosis, care goals.

Age was defined at the time of cohort entry based on date of birth and was categorized into the following groups: 0–1 month, 2–11 months, 1–9 years, 10–18 years, and older than 18 years of age. Migration background was defined as follows: the child immigrated into Germany and at least one parent was born in a country other than Germany, both parents immigrated into Germany, or neither parent had German citizenship [6].

The principal underlying diagnoses were categorized by the principal investigator, who is a pediatrician in the subspecialty palliative care. Categories for diagnoses were divided first into cancer and non-cancer and second into solid tumor, brain tumor, leukemia, neuromuscular (e.g., cerebral palsy), neurodegenerative, cardiovascular, respiratory, gastrointestinal, chromosomal aberration, and other conditions. The care goals were described by the SPPHC teams. The symptom prevalence and burden was assessed by the consulted SPPHC team. Permanent medication therapy or on-demand medication was retrieved from the medical chart.

### 2.2. Statistics

Descriptive statistics were used to characterize the study cohort. Comparisons of the two groups of cancer and non-cancer patients were computed using the *t*-test, the chi-square test, or Fisher’s exact test, depending on the variable analyzed. A two-tailed *p*-value of <0.05 was considered significant. For all analyses, Bonferroni adjustments were conducted. Statistical analyses were performed using IBM SPSS, version 22 (IBM, Chicago, IL, USA).

### 2.3. Ethics

The ethics committee of the University of Dresden, Germany approved the study. The approval code is EK 384122012; date of approval is 4 December 2012.

## 3. Results

Between April and September 2013, the nine SPPHC teams included *N* = 75 new patients into SPPHC.

### 3.1. Sample Characteristics

A total of 54 children (72%) suffered from non-oncologic conditions. The most common diagnoses were neuromuscular diseases (37%), followed by solid tumors (15%; see Table 1).

Characteristics of the study sample are summarized in Table 2. Gender was equally distributed within the sample. The median age of children was 7.7 years, ranging from 0 to 31 years (interquartile range = 13.1 years). A total of 10 (13.3%) patients were older than 18 years of age. Most patients lived with both parents (71%). Sixteen percent of patients (12 of 57) displayed a migration background. No significant differences could be found between the cancer and the non-cancer groups regarding gender, age, residence characteristics, and migration background.

### 3.2. Palliative Care Consultative Encounter Characteristics/Treatment Goals

In all cases, there was more than one care goal. For the majority of patients, counseling (93.2%) and symptom management (89.2%) were considered care goals. Additional care goals were empowerment (71.6%), communication about symptoms or about the disease course (68.9%), and decision-making support (64.9%). There were no significant differences between the cancer and the non-cancer group (see Table 3).

### 3.3. Symptoms

The most common symptoms when patients were admitted into SPPHC were impairment in communication, somatic pain, swallowing difficulties and cognitive impairment.

**Figure 1:**
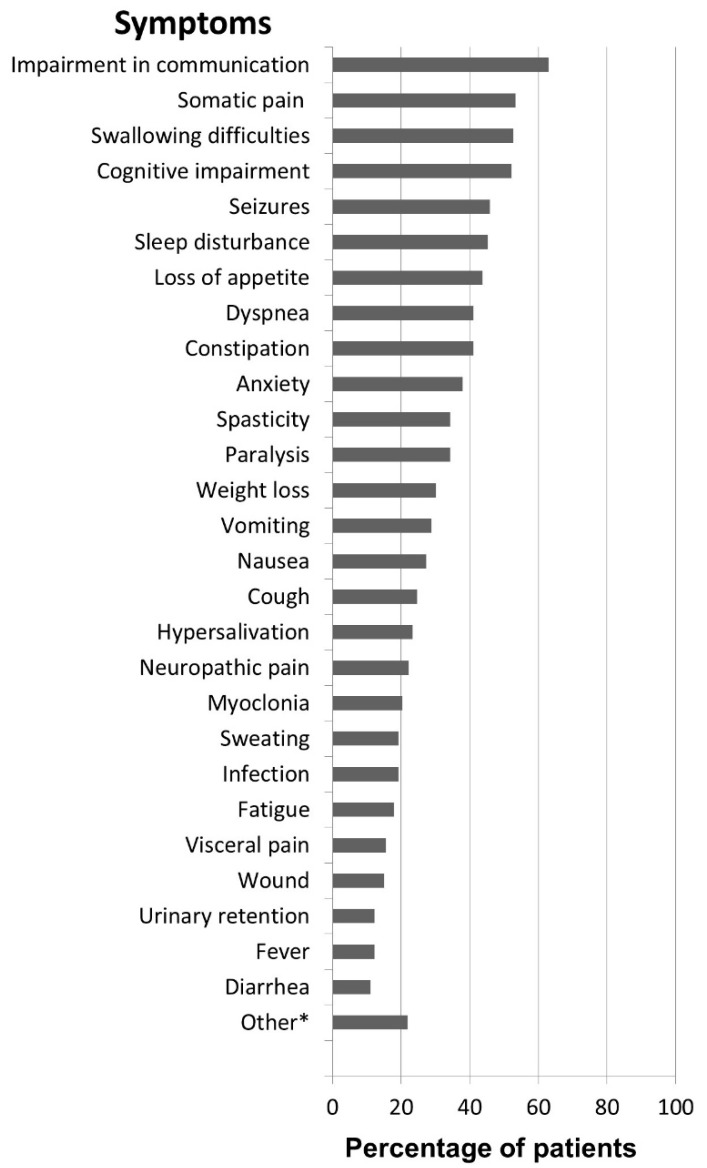
Symptom prevalence. * Other: hypothermia, dry mouth, edema, itching.

Patients suffering from pain (61%; *n* = 46) could be grouped into the following categories: suffering from somatic pain (85%), suffering from neuropathic pain (35%) and suffering from visceral pain (24%). The frequencies of all symptoms are shown in Figure 1.

The prevalence of specific symptoms differed between cancer and non-cancer patients. Swallowing difficulties, seizures and spasticity occurred significantly more in the non-cancer population (see Table 4).

**Figure 2:**
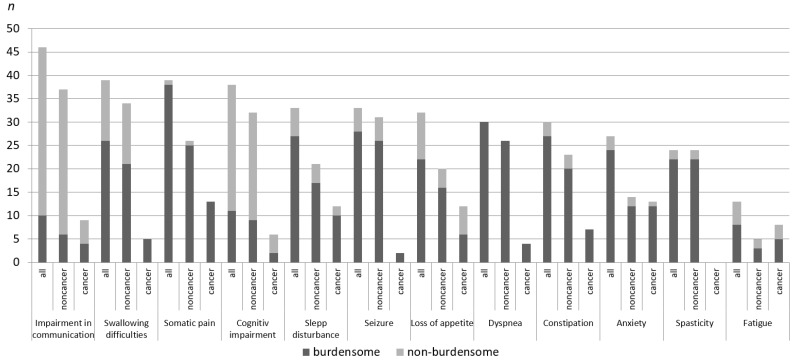
Symptom burden in patients with cancer and non-cancer condition.

The SPPHC teams also rated whether a symptom would be considered burdensome and needed treatment. Dyspnea was regarded as burdensome in all cases, and somatic pain was burdensome in all but one of the cases. In the cancer group, swallowing difficulties were considered a burdensome symptom in all cases, whereas this was only true for 15% of patients in the non-cancer group. Impairment in communication was classified as burdensome in 44% of patients (four of nine) in the cancer group and in 19% of patients (6 of 32) in the non-cancer group (see Figure 2).

### 3.4. Medication and Other Therapy

At the time of admission into SPPHC, approximately 57% of the patients had some type of feeding tube, of whom all but one were non-cancer patients (*p* < 0.001). Central venous catheters had been fitted in 20% of the patients, eleven of whom were cancer patients (*p* < 0.001). A tracheostomy tube had been attached to 9.3% of the patients, and a total of nearly 15% received invasive or non-invasive ventilation (8% and 6.7%, respectively). One child with severe cerebral palsy had an intrathecal Baclofen pump. All patients with tracheostomy or ventilation had a non-cancer condition. 

One third of the patients received oxygen, all of whom (except one) were non-cancer patients (*p* = 0.003). Children with non-cancer diagnoses received significantly more non-medication therapy than did those with cancer diagnoses (see Table 5).

Patients who were referred to SPPHC services received an average of close to four medications as permanent therapy and two medications as on-demand medication. Patients had a range of 0–12 medications for both categories (see Table 5).

The most common drugs used as permanent therapy were antacids (40% of all patients), followed by anticonvulsants (35.7%), and laxatives (32.9%). Non-opioids were the most common on-demand medications (38.6%), followed by sedatives and anticonvulsants (nearly 35%). Antiemetics were used significantly more often in the cancer group. For further information about medication therapy, see Table 6.

A total of 37.5% patients (27 of 72) received strong opioids, either as on-demand medication or as permanent therapy. The primary indications for strong opioids were pain (15 of 27) and dyspnea (10 of 27). Of all 46 patients with pain, twenty patients received strong opioids, fifteen of whom used them as permanent therapy. Seventy-four percent of patients suffering from pain received some type of analgesic as permanent therapy or as on-demand medication.

## 4. Discussion

The aim of this study was to prospectively describe patients referred to SPPHC teams in Germany. Furthermore, this study aimed to identify differences between non-cancer and cancer patients.

### 4.1. The “Typical” Pediatric Palliative Patient

Characteristics of the pediatric palliative patients in this study are heterogeneous and correspond to those identified in former studies [5,7,8]. A one-center study from Germany describes similar characteristics regarding the frequency of non-cancer conditions, age range, symptoms, and medications [5]. In a study by Feudtner and colleagues, which was conducted using a cohort drawn from Canada and the United States of America, similar results were reported [7]. Similarities can especially be found regarding the fitting of feeding tubes, central venous catheters, invasive and non-invasive ventilators, and tracheostomy tubes [7]. The most frequent care goals in the current study have been counseling (93.2%) and symptom management (89.2%). Relating burdensome symptoms and the treatment children received at referral indicates that symptom management in primary and secondary care might have not been sufficient (e.g., 25% of the patients with pain were not treated with analgesics). This is comparable with the results of Feudtner et al. [7], who identified symptom management, communication, and decision-making support as main care goals.

### 4.2. Cancer versus Non-Cancer Patients in Pediatric Palliative Care

Significant differences were identified between cancer and non-cancer patients. Non-cancer patients showed significantly higher rates of swallowing difficulties, spasticity and seizures. While cancer patients displayed a significantly higher rate of being treated with antiemetics and being fitted with a central venous catheter, non-cancer patients were especially fitted with a feeding tube and were treated with non-medication therapies and oxygen. It was only non-cancer patients who were attached to a ventilator or a tracheostomy tube.

The care goals developed by the SPPHC teams did not differ between the groups. Significant differences did exist, however, within the subgroups of the care categories counsel and empowerment. In the cancer group, the patients themselves required significantly more counseling and empowerment, while in the non-cancer group, the nursing services needed significantly more counseling. This discrepancy is likely due to the high rate of cognitive impairment within the non-cancer group.

In general, the existing care situation for non-cancer patients seems to be more complex upon admission by an SPPHC team and in 14% of the families more than one child is affected by a life-limiting condition. The patients more often received life-prolonging measures, such as being attached to a ventilator or a feeding tube. Other studies have also shown that non-cancer pediatric patients remain in palliative care longer than cancer patients [8]. Patients with earlier mortality within palliative care more often belong to the cancer group [7]. Children with complex, life-limiting conditions who are referred to a palliative care service, as well as their families, commonly verbalize goals related to health maintenance and independence [9]. These results indicate that care goals in pediatric palliative care are focused not only on end-of-life care but also on life-prolonging measures, such as feeding patients with a feeding tube. However, since the current study did not investigate characteristics at several time points, but rather collected the data only at the time of admission into palliative care, no conclusion can be drawn regarding the course and length of palliative care measures or related mortality.

Both patient groups displayed high demands for the management of symptoms; however, the type and number of symptoms differed. While communication impairments required treatment in 45% of patients in the cancer group, this was the case for only 19% of patients in the non-cancer group. Swallowing difficulties required treatment in all cases in the cancer group, while this symptom required treatment in only 64% of patients in the non-cancer group. Thus, depending on the diagnosis and the current treatment situation, the urgency for treating symptoms may differ.

With regard to care goals and the expected remaining survival time, different measures should be considered. A child suffering from a neuromuscular or neurodegenerative disease who also has swallowing difficulties does not necessarily require any interventions if he or she has already been fitted with a gastro- or jejunostomy tube. A similar child already fitted with a nasogastric tube may need to be fed with a gastro- or jejunostomy tube. A child suffering from an oncologic illness, in which survival time is estimated to be short, the care could include measures such as oral care, temporary provision of nutrients via a nasogastric tube, or parenteral supply of liquids.

### 4.3. The Pediatric Patient Compared with Adult Patients in Palliative Care

Recent studies have shown that pediatric patients differ significantly from adult patients in terms of the spectrum of diagnoses, the number of various symptoms, and the therapeutic measures taken [5].

While adult non-cancer patients account for only 8–23% of the cases [4,10,11], the proportion of non-oncologic cases of children varies from 70 to 80% [5,7,8]. The most common diagnoses of the non-oncologic group in adults include illnesses affecting the nervous system, heart (e.g., heart failure) and lungs (e.g., chronic obstructive pulmonary disease) [5,11]. Symptoms that appear in most adult cases include tiredness, fatigue, pain and the loss of appetite [5]. This range of symptoms appears to be comparable with that of the oncologic pediatric group in this study and previous studies with pediatric oncology patients [12,13]. The most common symptoms found in the current study, namely, communication impairment, swallowing difficulties, cognitive impairment, seizures, and sleeping difficulties, are of neurological origin.

### 4.4. Limitations

The study has several limitations. First, given the relatively small sample size at each of the nine sites, the data were not analyzed by site, although each site may have had differences in patient populations and consultative practices. However, the sample size is rather typical for studies conducted in pediatric palliative care. Second, symptoms were assessed only by a non-validated proxy-rating provided by care takers. It was not assessed with a validated measure provided by self-reports or parent proxy-reports. Third, this was a cross-sectional study and information on the patients’ course of disease, treatment process, and outcome were not assessed.

## 5. Conclusions

Patients who are referred to SPPHC teams in Germany are a diverse cohort, often with complex conditions. This complexity is characterized by a large range of differing diagnoses, heterogeneous, and mostly neurologically originating symptoms, and care goals that are focused on prolonging life. To address these goals, teams must be comprised of multiple professionals with different areas of expertise, especially neuropediatrics. The small number of pediatric cases limits the possibilities for conducting controlled studies and, therefore, developing evidence-based treatment guidelines. To overcome these difficulties and ensure high quality palliative care for children, each therapeutic measure must be conducted in close collaboration between the relevant pediatric departments.

## Figures and Tables

**Table 1:** Diagnoses of patients in the cohort.

Diagnoses (*N* = 75)	*n*	%
**Cancer**	21	28.1
Solid tumor	11	14.7
Brain tumor	8	10.7
Leukemia	2	2.7
**Non-Cancer**	54	71.9
Neuromuscular	28	37.0
Neurodegenerative	9	12.0
Chromosomal Aberration	6	8.0
Cardiovascular	6	8.0
Respiratory	1	1.3
Gastrointestinal	1	1.3
Other ^a^	3	4.0

^a^ VACTERL association (vertebral defects, anal atresia, cardiac defects, tracheo-esophageal fistula, renal anomalies, and limb abnormalities), agenesis of the pons, Lennox encephalopathy.

**Table 2:** Patient characteristics.

Characteristics	Total Patients		Cancer Patients		Non-Cancer Patients		Statistics		
	*n*	%	*n*	%	*n*	%	*df*	Chi-Square	*p*-Value
**Gender (*n* = 69)**							1	0.006	1.000
Female	34	49.3	10	50.0	24	49.0			
Male	35	50.7	10	50.0	25	51.0			
**Age (*N* = 75)**							4	7.45	0.114
0–1 month	1	1.3	0	0.0	1	1.9			
2–11 month	15	20.0	1	4.8	14	25.9			
1–9 years	27	36.0	8	38.1	19	35.2			
10–18 years	22	29.3	10	47.6	12	22.2			
Older than 18 years	10	13.3	2	9.5	8	14.8			
**Migration background**							2	0.42	0.810
With migration background ^1^	12	16.0	3	20.0	9	21.4			
**Residence (*N* = 75)**							2	3.99	0.136
With both parents	53	70.7	17	81.0	36	66.6			
Only/mostly with mother	13	17.3	4	19.0	9	16.7			
Other ^2^	9	12	0	0.0	9	16.7			
**Siblings with LLC (*n* = 72)**							2	3.01	0.222
Siblings with LLC	7	9.7	0	0.00	7	100.0			

^1^ Definition [6]; ^2^ Includes grandmother, hospice for adults, hospice for children (*n* = 2), sheltered accommodation, five of nine patients are older than 18 years; LLC, life-limiting conditions; *df*, degrees of freedom.

**Table 3:** Care goals.

Characteristics	Total Patients (*n* = 74)		Cancer Patients (*n* = 21)		Non-Cancer Patients (*n* = 53)		
	*n*	%	*n*	%	*n*	%	*p*-Value ^c^
Counsel ^a^	69	93.2	19	90.5	50	94.3	0.618
Symptom management	66	89.2	20	95.2	46	86.8	0.427
Empowerment ^b^	53	71.6	13	61.9	40	75.5	0.264
Communication about symptoms/course of disease	51	68.9	16	76.2	35	66	0.578
Decision-making support	48	64.9	16	76.2	32	60.4	0.282
Transition to home	15	20.3	6	28.6	9	17.0	0.388
Logistic and coordination of care	34	45.9	10	47.6	24	45.3	1.000
Discuss DNAR order	34	45.9	11	52.4	23	43.4	0.606
Consultation of further provider/carer	25	33.8	8	38.1	17	32.1	0.786

^a^ Counseling of the patient (*n* = 24), the family (*n* = 62), the pediatrician (*n* = 34), the nursing service (*n* = 23), the health care service provider (*n* = 6). Counseling of the patient and the nursing service is significant between cancer and non-cancer group (<0.001); ^b^ Empowerment of the patient (*n* = 13), the family (*n* = 49), the pediatrician (*n* = 7), the nursing service (*n* = 17). Empowerment of the patient is significant between cancer and non-cancer group (*p* < 0.001); ^c^ Fisher-test; DNAR, do not attempt resuscitation.

**Table 4:** Symptoms in cancer and non-cancer patients.

	Total Patients *n* (%)	Cancer Patients *n* (%)	Non-Cancer Patients *n* (%)	*p*-Value ^a^
Impairment in communication	46 (63.0)	9 (42.9)	37 (71.2)	0.033
Swallowing difficulties	39 (52.7)	5 (23.8)	34 (66.0)	0.002 *
Somatic pain	39 (53.4)	13 (61.9)	26 (50.0)	0.441
Cognitive impairment	38 (52.1)	6 (28.6)	32 (61.5)	0.019
Seizures	33 (45.8)	2 (9.5)	31 (60.8)	<0.001 *
Sleep disturbance	33 (45.2)	12 (57.1)	21 (40.4)	0.207
Loss of appetite	32 (43.8)	12 (57.1)	20 (38.5)	0.194
Constipation	30 (41.1)	7 (33.3)	23 (44.2)	0.441
Dyspnea	30 (41.1)	4 (19.0)	26 (50.0)	0.019
Anxiety	27 (38.0)	13 (65.0)	14 (27.5)	0.006
Paralysis	25 (34.2)	7 (33.3)	18 (34.6)	1.000
Spasticity	25 (34.2)	0 (0.0)	25 (48.1)	<0.001 *
Weight loss	22 (30.1)	8 (38.1)	14 (26.9)	0.403
Vomiting	21 (28.8)	8 (38.1)	13 (25.0)	0.271
Nausea	20 (27.4)	8 (38.1)	12 (23.1)	0.248
Cough	18 (24.7)	3 (14.3)	15 (28.8)	0.241
Hypersalivation	17 (23.3)	1 (4.8)	16 (30.8)	0.017
Neuropathic pain	16 (22.2)	3 (14.3)	13 (25.5)	0.365
Myoclonia	15 (20.3)	1 (4.8)	14 (26.4)	0.053
Infection	14 (19.2)	4 (19.0)	10 (19.2)	1.000
Sweating	14 (19.2)	1 (4.8)	13 (25.0)	0.092
Fatigue	13 (17.8)	8 (38.1)	5 (9.6)	0.007
Visceral pain	11 (15.5)	4 (19.0)	7 (14.0)	0.721
Wound	11 (15.1)	4 (19.0)	7 (13.5)	0.719
Urinary retention	9 (12.3)	4 (19.0)	5 (9.6)	0.269
Fever	9 (12.2)	5 (23.8)	4 (7.5)	0.107
Diarrhea	8 (11.0)	4 (19.0)	4 (7.7)	0.216
Other ^b^	16	5	11	

^a^ Fisher’s exact test; ^b^ Other: hypothermia, dry mouth, edema, itching; * result is significant after Bonferroni-Holm correction.

**Table 5:** Clinical characteristics of cancer and non-cancer patients.

Characteristics	All Patients		Cancer Patients		Non-Cancer Patients		
	Mean (SD)	Range	Mean (SD)	Range	Mean (SD)	Range	*p*-Value ^a^
**Number of medication (permanent therapy)**	3.80 (2.48)	0–12	3.33 (2.13)	0–7	4.00 (2.61)	0–12	0.305
**Number of medication (on demand medication)**	2.44 (2.02)	0–12	2.24 (1.58)	0–7	2.52 (2.19)	0–12	0.595
**Medical technology**	***n***	**%**	***n***	**%**	***n***	**%**	***p*-Value ^b^**
	75	100	21	100	54	100	
**Any feeding tube**	43	57.3	1	4.8	42	77.8	<0.001 *
Nasogastric tube	15	20.0	1	4.8	14	25.9	
Gastro-or jejunost. tube	29	38.7	0	0.0	29	53.7	
**Central venous catheter**	15	20.0	11	52.4	4	7.4	<0.001 *
**Tracheostomy**	7	9.3	0	0.0	7	13.0	0.180
**Any ventilation**	11	14.7	0	0.0	11	20.4	0.028
Invasive ventilation	6	8.0	0	0.0	6	11.1	
Noninvasive ventilation	5	6.7	0	0.0	5	9.3	
**Oxygen**	25	33.3	1	4.8	24	44.4	0.003 *
**VP/VA shunt**	3	4.0	2	9.5	1	1.9	0.188
**Other therapy than medication**	51	68.0	9	42.9	42	77.8	0.008 *
Physiotherapy	49	65.3	8	38.1	41	75.9	
Occupational therapy	18	24.0	2	9.5	16	29.6	
Speech therapy	10	13.3	0	0.0	10	18.5	
Music therapy	5	6.7	0	0.0	5	9.3	
Homeopathy	5	6.7	1	4.8	4	7.4	
Art therapy	1	1.3	0	0.0	1	1.9	
Acupuncture	0	0.0					

^a^*t*-Test; ^b^ Fisher’s exact test; * result is significant after Bonferroni-Holm correction; SD, standard deviation; VP/VA, ventriculoperitoneal/ventriculoatrial.

**Table 6:** Medication of cancer and non-cancer patients.

	All Patients		Cancer Patients		Non-Cancer Patients		
	*n*	%	*n*	%	*n*	%	*p*-Value ^a^
Permanent medication	70	100	21	100	49	100	
Antacids	28	40	9	42.9	19	38.8	0.794
Anticonvulsants	25	35.7	4	19.0	21	42.9	0.064
Laxatives	23	32.9	6	28.6	17	34.7	0.783
Immunotherapeutic	18	25.7	5	23.8	13	26.2	1.000
Vitamin	17	24.3	1	4.8	16	32.7	0.014
Strong Opioid	15	21.4	8	38.1	7	14.3	0.053
Antibiotics	15	21.4	6	28.6	9	18.4	0.356
Broncholytics	12	17.1	1	4.8	11	22.4	0.092
Non-Opioid	11	15.7	5	23.8	6	12.2	0.286
Muscle Relaxants	11	15.7	0	0.0	11	22.4	0.027
Antiemetics	7	10.0	6	28.6	1	2.0	0.002 *
Diuretics	7	10.0	0	0.0	7	14.3	0.094
Sedatives	4	5.7	1	4.8	3	6.1	1.000
Mild Opioids	3	4.3	1	4.8	2	4.1	1.000
Antidepressives	1	1.4	1	4.8	0	0.0	0.300
On demand medication	70	100	21	100	49	100	
Non-Opioid	27	38.6	7	33.3	20	40.8	0.603
Sedatives	24	34.3	5	23.8	19	38.8	0.280
Anticonvulsants	24	33.8	4	19.0	20	40.0	0.106
Strong Opioid	22	31.0	11	52.4	11	22.0	0.023
Laxatives	16	22.9	4	19.0	12	24.5	0.761
Antiemetics	15	21.4	10	47.6	5	10.2	0.001 *
Mild Opioid	4	5.7	1	4.8	3	6.1	1.000

^a^ Chi-square test; * result is significant after Bonferroni-Holm correction.
